# The Adult Film Industry: Time to Regulate?

**DOI:** 10.1371/journal.pmed.0040126

**Published:** 2007-06-19

**Authors:** Corita R Grudzen, Peter R Kerndt

## Abstract

The industry is putting the health of its employees, adult film perfomers, at risk by insisting that they work without condoms.

The United States adult film industry produces 4,000–11,000 films and earns an estimated $9–$13 billion in gross revenues annually [1]. An estimated 200 production companies employ 1,200–1,500 performers [2]. Performers typically earn $400–$1,000 per shoot and are not compensated based on distribution or sales.

Los Angeles County is the largest center for adult film production worldwide. In 1988 the California Supreme Court, in *People v. Freeman*, found adult film production to be protected as free speech under the First Amendment, since such films were not considered obscene based on prevailing community standards. Unlike other legal but highly regulated activities such as gambling and commercial sex work in Nevada, the adult film industry was legalized in California through case law, not by statute, and has for the most part escaped governmental oversight. Regulation of the industry has been limited to prevention of child pornography. Title 18, Section 2257 of the United States Code of Regulations explicitly prohibits performers under age 18 and provides for civil and criminal prosecutions for any violation [3]. Adult film production companies are required to have a Custodian of Records to document and retain records of the age of all performers, to enforce the age entry restriction.

Adult film performers engage in prolonged and repeated sexual acts with multiple sexual partners over short periods of time, creating ideal conditions for transmission of HIV and other sexually transmitted diseases (STDs). All the more concerning, high-risk practices are on the rise [4]. These practices include sex acts that involve simultaneous double penetration (double-anal and vaginal–anal intercourse) and repeated facial ejaculations. At the same time, condom use is reportedly low in heterosexual adult films—approximately 17% for adult performers [5]. In 2004, only two of the 200 adult film companies required the use of condoms for all penile–anal and penile–vaginal penetration [2]. Performers report that they are required to work without condoms to maintain employment.

These practices lead to high transmission rates of STDs and occasionally HIV among performers. After four performers contracted HIV in 1998, Sharon Mitchell, a former adult film performer, founded Adult Industry Medical (http://www.aim-med.org), a clinic to counsel and screen performers monthly for HIV using a PCR test (Figure 1). It was expanded later to include other STD testing. The testing program began as an effort to reduce transmission of infections through early diagnosis, treatment, and “quarantine” should a performer test positive for HIV. Performers are required in most cases to pay for all screening tests, and to sign a consent form that permits disclosure of their test results to other performers and producers before filming. Both of these practices are explicitly prohibited under California Occupational Safety and Health Administration (Cal/OSHA) regulations. HIV-positive female performers are permanently excluded from participating in adult films.

**Figure 1:**
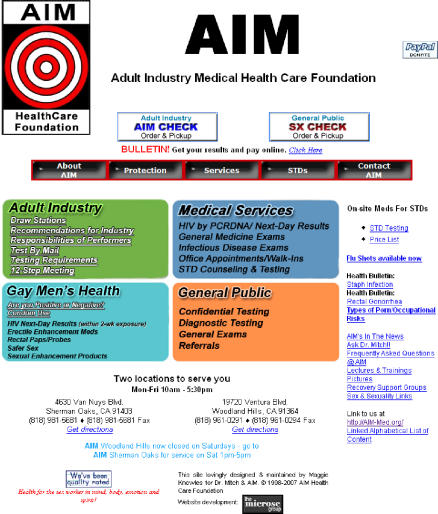
Screenshot of the Web site of Adult Industry Medical Health Care Foundation

## Worker Safety and Public Health

The current practice of periodic HIV and STD testing may detect some disease early, but often fails to prevent transmission. The most recent HIV outbreak occurred when three performers who had been compliant with monthly screening contracted HIV in April of 2004 [6]. At that time, a male performer who had tested HIV negative only three days earlier infected three of 14 female performers.

Other STDs are also highly prevalent in the industry. Among 825 performers screened in 2000–2001, 7.7% of females and 5.5% of males had chlamydia, and 2% overall had gonorrhea [7]. These rates are much higher than in patients visiting family planning clinics, where chlamydia and gonorrhea rates were 4.0% and 0.7%, respectively [8]. Some might argue that this program of STD testing keeps rates of HIV and other STDs lower than in other sex-related industries, and in fact, a recent study of prostitutes in San Francisco found 6.8% and 12.4% positivity rates for chlamydia and gonorrhea, higher than rates in the adult film industry [9].

Between January 2003 and March 2005, approximately 976 performers were reported with 1,153 positive STD test results. Of the 1,153 positive test results, 722 (62.6 %) were chlamydia, 355 (30.8%) were gonorrhea, and 126 (10.9%) were coinfections with chlamydia and gonorrhea [10]. Less is known about the prevalence and risk of transmission of other STDs such as syphilis, herpes simplex virus, human papillomavirus, hepatitis B or C, trichomonal infection, or diseases transmitted through the fecal–oral route.

Efforts to reduce the risk of HIV and other STD transmission must include the use of condoms. Even with the PCR testing currently used within the industry, a recently infected performer can test negative during the window in which they are highly infectious and go on to transmit the virus to others. A meta-analysis suggests that condoms are 90%–95% effective in preventing HIV transmission [11]. Condoms are especially important given the high-risk sex acts increasingly being performed in the industry. When looking at HIV exposure risks by site, receptive anal sex has the highest risk at 80 instances of transmission per 10,000 exposures [12], higher than needle stick injuries (10–50 per 10,000) [13] or receptive vaginal penetration (10 per 10,000) [14]. Pre-existing infection with other STDs also increases the risk of HIV transmission. One study showed that the relative risk of HIV acquisition in a vaginal receptive partner increases 2- to 4-fold when the receptive partner is infected with herpes simplex type 2 [15].

Performers may also be exposed to HIV and other STDs outside the workplace. Performers may be engaged in commercial sex work through escort services or use intravenous drugs, risking HIV and hepatitis C infection. The use of condoms would prevent performers who had acquired HIV and STDs outside the workplace from transmitting these infections to other performers in the workplace. Additionally, condoms would help prevent unwanted pregnancy and the complications of STDs, which include ectopic pregnancy, pelvic inflammatory disease, and infertility. Little is currently known about the prevalence of these diseases in performers.

The portrayal of unsafe sex in adult films may also influence viewer behavior. In the same way that images of smoking in films romanticize tobacco use, viewers of these adult films may idealize unprotected sex [16]. The increasingly high-risk sexual behavior viewed by large audiences on television and the Internet could decrease condom use. Requiring condoms may influence viewers to see them as normative or even sexually appealing, and devalue unsafe sex. With the growing accessibility of adult film to mainstream America, portrayals of condom use onscreen could increase condom use among viewers, thereby promoting public health.

In contrast to heterosexual adult films, homosexual-targeted productions more consistently require condoms. Due to the large number of HIV-positive performers, there is no requirement for HIV testing and condom use is the norm. Despite the ubiquitous use of condoms, homosexual adult movies are popular and profitable for production companies. In fact, there is some evidence that homosexual male audiences would not tolerate movies with unsafe sex, likely due to their proximity to many with HIV in the homosexual community. Some homosexual audiences regard watching sex without condoms as “watching death on the screen” [16].

## Regulation of Sex-Related Industries

Legislators can look to Nevada for a model for the successful regulation of a legal sex-related industry. Since the institution of mandatory condoms in Nevada's brothels in 1988, not a single sex worker has contracted HIV [17]. Workers must be repeatedly tested for HIV, syphilis, gonorrhea, and chlamydia to maintain a state health and work card. There are numerous other international models for condom enforcement in sex work, from Mexico City to Amsterdam. While there is no clear model for mandatory condom use in adult film, Brazil boasts an 80% condom usage rate in their adult films [18], while still maintaining a large share of the international market as the world's second largest adult film industry [18]. This suggests that condom use in adult films does not have to erode profitability. It is also possible to use filming techniques to reduce the visual effect of condoms, by using flesh tone–colored condoms or by digitally removing them post-production. Facial ejaculations could be simulated through the use of inert materials such as liquid antacids combined with filming techniques, which would eliminate any health risk to the performer.

Vivid Entertainment Group, one of the largest producers of adult film in the US, temporarily implemented a condom-only policy after the HIV outbreak in 2004 but has since reversed this company policy. Although some companies may voluntarily decide to be condom-only, it is unlikely that this industry will establish safer working conditions for employees without external regulation. A state or national mandate would level the playing field for all companies and not give an unfair advantage to those who decide to produce films without condoms.

## Occupational Health and Safety

In California, every employer is required to ensure that employees have a safe working environment. In 1973, the California Occupational Safety and Health Act was enacted to assure “safe and healthful working conditions for all California working men and women by authorizing the enforcement of effective standards, assisting and encouraging employers to maintain safe and healthful working conditions, and by providing for research, information, education, training, and enforcement in the field of occupational safety and health” [19]. Each employer must establish, implement, and maintain a written Injury and Illness Prevention Program according to Title 8 of the State Code of Regulations [20]. This includes components for training programs and disciplinary actions. Employers must protect employees from blood-borne pathogens and not discriminate against employees that complain about safety and health conditions. Companies are required to prevent workers from coming into contact with blood or other potentially infectious material, including semen and vaginal fluid, and to provide post-exposure prophylaxis. Universal precautions, which assume all material is potentially infectious, are part of the blood-borne pathogens standard.

In the health care setting, it is hard to imagine a clinic or hospital not providing and requiring its employees to wear gloves or other personal protective equipment. If a health care worker has a needle stick or other potentially infectious fluid exposure on the job, systems are in place to rapidly and effectively treat the employee to prevent transmission of HIV and other infectious diseases. Although a legal industry, adult film has allowed consistent exposure of its employees to HIV, hepatitis, human papillomavirus, herpes simplex virus, chlamydia, gonorrhea, and other diseases without liability or worker recourse.

Cal/OSHA has recently made recommendations specific to adult film to protect performers from acquiring sexually transmitted infections [21]. This includes the use of personal protective equipment (condoms and dental dams) as barriers, simulation of sex acts post-production, and ejaculation outside the partner's body. In addition, post-exposure prophylaxis after possible exposure to pathogens such as hepatitis B and HIV would be required. This would greatly reduce transmission of HIV and other STDs and would likely prevent transmission in cases where a screening test does not detect an infected performer. Cal/OSHA also requires a procedure for exposure incidents when an employee has contact with potentially infectious material. The employer must provide a medical evaluation and follow-up at no cost to the employee. The final component is a requirement that each employee receive training about blood-borne pathogens, including how they can protect themselves against infection and what to do if they are exposed.

## Attempts at External Regulation

Mandatory reporting in California is required for chlamydia, gonorrhea, HIV, syphilis, chancroid, non-chlamydial non-gonorrheal urethritis, and pelvic inflammatory disease. The Los Angeles County Department of Public Health has monitored the industry to assure that performers receive adequate treatment and follow-up for STDs and has endorsed external regulation of the industry that would require condom use, STD screening, and education to prevent STD transmission.

Recognizing that local regulations would have limited impact and seeking to establish existing standards for work health and safety in the industry, officials from the Los Angeles Department of Public Health requested an investigation of the April 2004 HIV outbreak. In September of 2004, Cal/OSHA fined the two production companies in the outbreak $30,560 each for failure to comply with blood-borne pathogen standards [22]. Having established that regulation does apply to the industry, enforcement of the workplace standards is now the issue. OSHA is limited by the number of enforcement officials and therefore will only act in response to a complaint. Workers may be unaware of their rights or reluctant to file a complaint for fear of loss of employment or employer retaliation.

Response from California legislators has been limited. In June of 2004, Assemblyman Paul Koretz, Chair of the Assembly Committee on Labor and Employment, organized an informational hearing in the San Fernando Valley to consider the feasibility and potential impact of mandating HIV/STD screening and condom use. The hearing, entitled, “Worker Health and Safety in the Adult Film Industry,” drew together officials from Cal/OSHA, the Los Angeles Department of Public Health, the California Department of Health Services, the American Civil Liberties Union, and the industry trade organization, Free Speech Coalition [23]. In response to the hearing, Assemblyman Koretz sent a letter to 185 adult film production companies urging them to adopt condoms or face legislative action [24].

Two years later, this letter has had little to no effect and the adult film industry continues to produce the great majority of films without condoms. In October of this year, a multi-stakeholder meeting was convened at the University of California to readdress the issue of worker safety. A group of 65 participants including performers, industry executives, state and local health officials, and legal representatives spent the day debating the controversies and difficulties of mandated STD screening and condom regulation. Concerns were raised about the industry going underground or moving out of state should there be a state but no national requirement. Many present felt it would be difficult to regulate small production companies that distribute their films primarily via the Internet. There was an emphasis on the need for a multi-faceted solution that involves the extension of existing worker protection to this industry with better enforcement, the organization and potential unionization of performers, increased public awareness, and thoughtful legislation (Box 1).

**Box 1. Potential Policy Changes**

- National legislation that includes regulation of internet-based adult films
- Mandatory condom use with condom seal of approval
- Film rating system based on set safety criteria
- Licensure of performers
- STD testing paid for by the industry
- Vaccinations against human papillomavirus and hepatitis B and post-exposure prophylaxis paid for by the industry
- Education and training of all workers and employees
- Legal age of performers raised from 18 to 21 years old
- Drug testing of performers

## The Future

Lacking the will or ability to regulate itself, the adult film industry needs state and federal legislation to enforce health and safety standards for adult film performers. Local officials lack the authority to impose fines and Cal/OSHA's monitoring and enforcement capability is limited. Short of legislation mandating performer protection, restricting distribution of adult movies to condom-only films may be the one way to have an impact on the industry. If there were organized and truly effective advocacy for performers, then large hotel chains, video retailers, and cable networks could be pressured to purchase adult films under a condom-only “seal of approval.” Alternatively and more effectively, legislation could require that the Custodian of Records (already required under Federal law) maintain documentation of screening tests and condom usage in a film's production. Distribution could be restricted to those films produced pursuant to the standard prior to any sale to cable companies or hotel chains, over the Internet, or in other markets.

While some argue that adult film will go underground if condoms become mandatory, it is hard to imagine that a legal multi-billion dollar industry would disappear. Distributors and production companies have become so entrenched in Southern California that it seems unlikely that they would move to another location or go clandestine. Adult film is now so accepted and widespread that it cannot easily escape regulation, especially now that is so readily accessible on the Internet, cable networks, and in most major hotels. Unfortunately, the growing popularity of adult film has not translated into safer working conditions for performers. It is unethical for industry executives, legislators, and consumers to continue to enjoy the profits, tax revenues, and gratification of adult film without ensuring the safety of performers.

## Abbreviations

Cal/OSHA: California Occupational Safety and Health Administration
STDs: sexually transmitted diseases
